# Reduced top‐down attentional control in adolescents with generalized anxiety disorder

**DOI:** 10.1002/brb3.1994

**Published:** 2020-12-25

**Authors:** Johannah Bashford‐Largo, Joseph Aloi, Jennie Lukoff, Kimberly Johnson, Stuart F. White, Matthew Dobbertin, Robert James Blair, Karina S. Blair

**Affiliations:** ^1^ Center for Neurobehavioral Research Boys Town National Research Hospital Omaha NE USA; ^2^ Department of Psychiatry Indiana University School of Medicine Indianapolis IN USA

**Keywords:** anxiety disorders, attention, brain imaging/neuroimaging, functional MRI, GAD/generalized anxiety disorder

## Abstract

**Background:**

Generalized anxiety disorder (GAD) can significantly impair quality of life and is associated with a relatively poor long‐term prognosis. Anxiety disorders are often associated with hyper‐responsiveness to threat, perhaps coupled with impaired executive functioning. However, GAD, particularly adolescent GAD, has been the focus of little functional neuroimaging work compared to other anxiety disorders. Here, we examine the neural association of adolescent GAD with responsiveness to threat and response control.

**Methods:**

The study involved 35 adolescents with GAD and 34 healthy comparison individuals (*N* = 69) matched on age, gender, and IQ. Participants were scanned during an affective number Stroop task.

**Results:**

We found significant Group‐by‐Task Condition interactions in regions involved in response control/motor responding (bilateral precentral gyri and cerebellum) and/or cognitive control/attention (dorsomedial and lateral frontal cortex, posterior cingulate cortex, cuneus, and precuneus). In line with predictions, the youth with GAD showed significantly less recruitment during task trials than the healthy comparison individuals. However, no indications of specific heightened responses to threat were seen.

**Conclusions:**

GAD involves reduced capacity for engaging regions involved in response control/motor responding and/or cognitive control/attention. This might reflect either a secondary consequence of the patient's worry or an early risk factor for the development of worry.

## Graphical abstract

Generalized anxiety disorder (GAD) can significantly impair quality of life and is associated with a relatively poor long‐term prognosis. However, GAD, particularly adolescent GAD has been the focus of little functional neuro‐imaging work compared to other anxiety disorders. Here we examine the neural association of adolescent GAD with responsiveness to threat and response control.

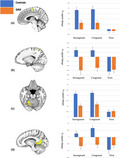

## 1 INTRODUCTION

Generalized anxiety disorder (GAD) is an anxiety disorder where the patient's excessive worry and anxiety significantly impair quality of life. Symptoms of GAD include restlessness, fatigue, irritability, and sleep disturbance (DSM, American Psychiatric Association, 2013). GAD is common in adolescence and is associated with a relatively poor long‐term prognosis (Pine et al., 1998). However, despite this, GAD, and particularly adolescent GAD, has been the focus of relatively little functional neuroimaging work.

A core feature of most anxiety disorders is hyper‐responsiveness to threat. For example, both adolescents and adults with social anxiety disorder show increased responsiveness to social “threats” within the amygdala and connected cortical structures (e.g., Blair et al., 2008, 2016; Gentili et al., 2016; Pine, 2001). Surprisingly, the literature is less clear cut with respect to GAD (for a review, see Fonzo & Etkin, 2017). There have been reports that adult patients with GAD, relative to comparison healthy individuals, show increased (e.g., Buff et al., 2017), comparable (e.g., Whalen et al., 2008) or decreased responsiveness (e.g., Blair et al., 2008, 2012). Findings with pediatric cases of GAD, though rarer, have been more consistent but indicate contextual importance (Fonzo & Etkin, 2017). For example, one study reported amygdala hyperactivity to faces but only if the participant was attending to their own subjective feeling of fear in response to the stimulus, rather than simply attending to the stimulus (McClure et al., 2007).

Potentially related to this literature on emotional responding, there have been suggestions that patients with GAD face difficulties in actively downregulating emotional responses to affective stimuli (Decker et al., 2008; Mennin et al., 2005; Patriquin & Mathew, 2017). Active downregulation via cognitive reappraisal is thought to involve the volitional recruitment of regions implicated in top‐down attention (dorsomedial, lateral frontal [dmFC and dlFC] and parietal cortices; Braunstein et al., 2017; Buhle et al., 2014). Consistent with this, two studies with adults with GAD have reported reduced recruitment of dmPFC and either lateral frontal or parietal cortices during cognitive reappraisal of emotional stimuli (Ball et al., 2013; Blair et al., 2012). Reduced emotional responding to *emotional distracters* is also seen as a result of volitional recruitment of regions implicated in top‐down attention to task‐related stimuli (Blair et al., 2007; Mitchell et al., 2008; Pessoa et al., 2002). Adult*s* with GAD also show reduced recruitment of dmPFC and parietal cortices in response to task‐related stimuli in these paradigms and they do so during both implicit and explicit task instructions (Blair et al., 2012). However, the recruitment of top‐down attentional regions during either cognitive reappraisal or task‐related processing in the presence of emotional distracters has not, to our knowledge, been investigated in *adolescents* with GAD.

**Figure 1:**
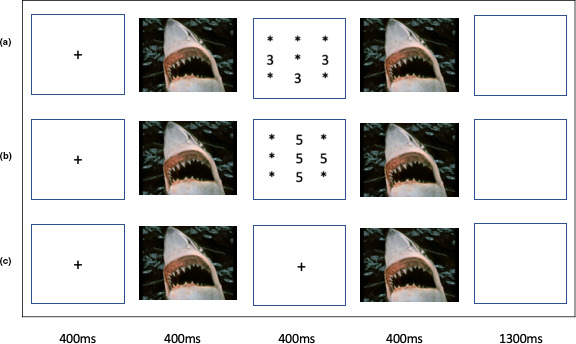
Task illustration. Example of (a) Negative Congruent, (b) Negative Incongruent, and (c) Negative View trial

The goal of the current study was to investigate the responsiveness of neural systems engaged in responding to emotional stimuli, response control, and top‐down attention during task‐related processing in the presence of emotional distracters in *adolescents* with GAD. Thirty‐five adolescents with GAD and a matched comparison group of 34 typically developing adolescents performed the Affective Number Stroop task (Blair et al., 2007), a task used previously within adults with GAD (Blair et al., 2012). During performance of this task, participants either perform goal‐directed activity (counting the number of numerals) in the context of emotional or neutral distracters or simply process the emotional or neutral images (see Figure 1 **)**. The level of BOLD responses to negative relative to neutral stimuli allows an index of differential negative emotional reactivity at the neural level (i.e., the group‐by‐emotion interaction results). In line with previous work with adults with GAD on this task (Blair et al., 2012), we predicted the following: (i) adolescents with GAD would show evidence of: (i) reduced responsiveness to emotional stimuli (Group‐by‐Valence interaction) within the amygdala; and (ii) reduced recruitment of regions implicated in response control and top‐down attention during task performance (Group‐by‐Task Condition interaction), that is, within dorsomedial and parietal cortices.

## 2 METHODS AND MATERIALS

### 2.1 Participants

Thirty‐five adolescents with GAD and 34 healthy comparison individuals participated in the study. The two groups were matched on age, gender, and IQ (see Table 1). However, and consistent with the literature (Remes et al., 2018; Shen et al., 2018), 15 of the adolescents with GAD presented with comorbid major depressive disorder. Clinical characterization was done through psychiatric interviews by licensed and board‐certified psychiatrists with the participants and their parents, to adhere closely to common clinical practice.

**Table 1:** Subject characteristics

	GAD (*N* = 35)	No GAD (*N* = 34)	*p*<	Test statistic
Basic demographics				
Age	15.32 (*SD* = 1.75)	15.9 (*SD* = 1.47)	.100 (ns)	*t* = −1.668
Sex	18 F/17 M	14 F/20 M	.402 (ns)	*X* ^2^ = 0.703
IQ	105.59 (*SD* = 9.76)	103.43 (*SD* = 13.26)	.445 (ns)	*t* = 0.768
GAD SCARED score	12.0 (*SD* = 4.02)	2.9 (*SD* = 1.90)	<.001	*t* = −10.755
MDD	*N* = 15	–	–	
Antipsychotic medications	4 (11%)	–	–	
SSRIs	11 (31%)	–	–	
Stimulants	4 (11%)	–	–	

Note

Key to table.

Abbreviation: SCARED, Screen for Child Anxiety‐related Disorders.

Participants were recruited either shortly after their arrival at a residential care facility (Boys Town) or from the community. Youth recruited from the residential care facility were referred because of behavioral and mental health problems. The Boys Town youth are made up of participants with severe internalizing and externalizing pathology (sometimes co‐occurring in the same youth). The participants in this study represented participants with severe internalizing pathology. Moreover, Boys Town is home to both males and females, with approximately 40% of the residents being female and our sample reflects that (of the 35 participants with GAD from Boys Town in the current study, 51% were female). The BT intervention involves a psychosocial residential treatment model with clinical contact with psychiatrists, clinical psychologists, and educational psychologists. The model is heavily positive reinforcement‐based and set within a rural environment to further reduce anxiety/threat. Medications are administered if felt appropriate though attempts are made to wean clients off over‐prescription of medications that may have occurred preintake.

Community members were recruited via flyers. Participants were excluded if IQ was below 80 or if they had medical illnesses that required the use of medication that may have psychotropic effects, such as beta‐blockers or steroids. Medications provided for psychiatric disorders (specifically antipsychotic, stimulant, or mood‐stabilizing medications) were not exclusory but participants were asked to withhold medication on the day of the scan. Exclusion criteria also included braces, claustrophobia, active substance dependence (Boys Town routinely checks for substance use), pervasive developmental disorder, Tourette's syndrome, lifetime history of psychosis, neurological disorder, head trauma, non‐English speaking, and presence of active safety concerns.

A doctoral level researcher or a member of the clinical research team obtained written informed consent and assent. In all cases, youth had the right to decline participation at any time before or during the study. Consent documents were reviewed with the parent/legal guardians and written permission was obtained (1) at the initial visit for community participants or (2) at the time of intake for youth placed in Boys Town programs. Assent was obtained from the Boys Town youth in a separate session. In all cases, youth had the right to decline participation at any time before or during the study. It was made clear to all participants and their parents that their decision with respect to participation had no influence on their clinical care. The Boys Town National Research Hospital institutional review board approved this study.

### 2.2 Affective Number Stroop

The Affective Number Stroop task was adapted from prior work by our group (Blair et al., 2007, 2019; Hwang et al., 2016). The emotional stimuli consisted of 16 negative, 16 neutral, and 16 positive pictures selected from the International Affective Picture System (IAPS) (Lang & Greenwald, 1988). The individual cognitive task stimuli consisted of displays of numbers and the cognitive task involved deciding how many numbers were displayed in each display. Specifically, participants pressed button 3, 4, 5, or 6 to indicate whether there were 3, 4, 5, or 6 numbers in the display.

Each trial began with a fixation point presented in the middle of the screen for 400 ms. For trials involving a goal‐directed task (*task* trials), the fixation point was replaced by an image presented for 400 ms, followed by the numerical display presented for 400 ms, followed by the image presented for a further 400 ms, followed by a blank stimulus for 1,300 ms. On incongruent or difficult task trials, the Arabic numeral distracter information was inconsistent with the numerosity information (e.g., four 5 s; see Figure 1a). On congruent task trials, the Arabic numeral distracter information was consistent with the numerosity information; (e.g., four 4 s; see Figure 1b). For the view or no task trials (*view* trials; see Figure 1c), the numerical display was simply replaced by a fixation point.

There were two runs, each consisting of 16 presentations of each Valence‐by‐Task condition randomized throughout the run (i.e., 144 per run). In addition, 40 fixation points (staying on the screen for the duration of a condition trial 2,500 ms) were randomly presented throughout each run to serve as an implicit baseline. Thus, each participant was presented with 32 trials of each Valence‐by‐Task Condition across the two runs (288 task trial presentations, 80 fixation point trials).

### 2.3 Anxiety measures

*Screen for Child Anxiety Related Emotional Disorder* (SCARED, child version, Birmaher et al., 1997) is a self‐report questionnaire that looks at a youth's potential for having an anxiety disorder. There are 5 subsets including generalized anxiety disorder (9 questions) Panic Disorder (13 questions), Separation Anxiety Disorder (8 questions), Social Anxiety Disorder (4 questions), and School Anxiety (4 questions). Prior work has indicated that the SCARED has shown to have excellent internal consistency and test‐retest reliabilities (α= 0.921 and *r *= 0.782 for random effects model) (Stringaris et al., 2012).

### 2.4 fMRI parameters

Whole‐brain blood oxygen level‐dependent (BOLD) fMRI data were acquired using a 3.0 Tesla Siemens Skyra Magnetic Resonance Scanner. A total of 384 functional images were taken, divided over two runs, with a T2*‐weighted gradient echo‐planar imaging (EPI) sequence (repetition time (TR) = 2,500 ms, echo time (TE) = 27 ms, flip angle = 90°, field‐of‐view (FOV) = 240 mm). Whole‐brain coverage was obtained with 43 axial slices (thickness, 2.5 mm; voxel size 2.6 × 2.6 × 2.5 mm^3^; distance factor 21%). In the same session, a high‐resolution T1‐weighed anatomical image was acquired to aid with spatial normalization (MP‐RAGE, repetition time = 2,200 ms, echo time = 2.48 ms; 230 mm field‐of‐view; 8° flip angle; 256 × 208 matrix) was acquired to register with the EPI dataset. Whole‐brain coverage was obtained with 176 axial slices (thickness 1 mm; voxel size 0.9 × 0.9 × 1 mm^3^, distance factor 50%).

### 2.5 fMRI Analysis: Data preprocessing and individual‐level analysis

Functional MRI data were preprocessed and analyzed using Analysis of Functional NeuroImages (AFNI) software (Cox, 1996). Data from the first four repetitions were collected prior to magnetization equilibrium and were discarded. The anatomical scan for each participant was registered to the Talairach and Tournoux atlas (Talairach & Tournoux, 1988), and each participant's functional EPI data were registered to their Talairach anatomical scan in AFNI. Functional images were motion corrected and spatially smoothed with a 6‐mm full width half maximum Gaussian kernel. The data then underwent time series normalization and these results were multiplied by 100 for each voxel. Therefore, the resultant regression coefficients are representative of a percentage of signal change from the mean.

Following this, regressors depicting each of the response types were created by convolving the train of stimulus events with a gamma‐variate hemodynamic response function to account for the slow hemodynamic response. As such the model of the response began with trail onset and was modeled via a gamma‐variate hemodynamic response function. There was no feedback provided in this study (participants were not informed if their responses were correct during task performance—only during practice outside the scanner).

Ten regressors depicting each of the response types were created (Negative View, Negative Congruent, Negative Incongruent, Neutral View, Neutral Congruent, Neutral Incongruent, Positive View, Positive Congruent, Positive Incongruent, and Error/Missed Responses). Linear regression modeling was then performed using the regressors described above plus regressors to model a fourth‐order baseline drift function. This produced for each voxel and each regressor, a beta coefficient and its associated *t*‐statistic.

### 2.6 Statistical analyses performed

#### 2.6.1 Behavioral data

*Accuracy and reaction time (RT):* Accuracy and RT data were analyzed via two 2 (Group: Participants with GAD, participants without GAD) × 2 (Task Condition: Congruent, Incongruent) × 3 (Valence: Negative, Neutral, Positive) repeated measures ANOVAs.

#### 2.6.2 fMRI data

##### Movement data

Volumes were censored if there was >0.5 mm motion across adjacent volumes. No participant had more than 5% censored volumes. Potential group differences in movement were analyzed via three one‐way ANOVAs (for average motion per volume, censored volumes, and maximum displacement during scanning, respectively).

##### BOLD response data

We tested our hypotheses regarding GAD via a 2 (Group: Participants with GAD, participants without GAD) × 3 (Task Condition: Incongruent, Congruent, View) × 3 (Valence: Negative, Neutral, Positive) ANOVA conducted on the BOLD response data. To facilitate future meta‐analytic work, effect sizes (partial eta square [$\eta_{p}^{2}$]) are reported in the Tables.

##### Follow‐up analysis

Given the potential influences of medication use, the group‐based ANOVA above was rerun excluding participants using, respectively, stimulants, antidepressant, and antipsychotics. Given the comorbidity of GAD with MDD, the group‐based ANOVA above was rerun excluding participants with MDD.

##### Correction for multiple comparisons

We used a spatial clustering operation in AFNI's 3dClustSim utilizing the autocorrelation function (‐acf) with 10,000 Monte Carlo simulations for the whole‐brain analysis. Spatial autocorrelation was estimated from residuals from the individual‐level GLMs. The initial threshold was set at *p *= .001. This process yielded an extant threshold of *k* = 20 voxels for the whole brain (multiple comparison corrected *p* < .05).

## 3 RESULTS

### 3.1 Behavioral data

There were significant main effects of Task Condition for both accuracy and RT (*F*(1, 67) = 34.83 & 156.08 respectively; *p* = .002 & *p* < .001; $\eta_{p}^{2}$ = 0.342 & 0.700). As expected, participants were significantly more accurate and faster when responding to Congruent (*M*_ac_ = 86.25%, *M*_rt_ = 767.37 ms) relative to Incongruent trials (*M*_acc_ = 80.57%, *M*_rt_ = 827.63 ms), indicating that the task elicited the intended behavioral effects. There was also a significant main effect of group for error rates (*F*(1, 67) = 10.48; *p* < .05; $\eta_{p}^{2}$ = 0.139) with healthy participants making less errors than participants with GAD (*M*_HCacc_ = 88.42%; *M*_GADacc_ = 78.54%). In addition, there was a significant Group‐by‐Valence interaction for the RT data (*F*(2, 134) = 6.20; *p* = .003; $\eta_{p}^{2}$ = 0,085). Healthy participants showed a significantly greater increase in RT in the presence of positive relative to neutral distracters than participants with GAD (*t*(67) = 3.47; *p* = .001; the results were in the same direction for negative relative to neutral distracters but only at weak trend levels ‐ *t*(67) = 1.45; *p* = .152). There were no other significant effects involving group.

### 3.2 Movement data

Volumes were censored if there was >0.5 mm motion across adjacent volumes. No participant had >5% censored volumes. There were no significant group differences in terms of average motion per volume (*M*_GAD_ = 0.082 vs. *M*_HC_ = 0.083: *F* = 0.03; *p* = .865), censored volumes (*M*_GAD_ = 0.429 vs. *M*_HC_ = 1.088: *F* = 2.90; *p* = .093), or maximum displacement during scanning (*M*_GAD_ = 3.202 vs. *M*_HC_ = 3.322: *F* = 0.09; *p* = .767).

### 3.3 EPI data

We tested our hypotheses regarding GAD via a 2 (Group: Participants with GAD, participants without GAD) × 3 (Task Condition: Incongruent, Congruent, View) × 3 (Valence: Negative, Neutral, Positive) ANOVA. In line with predictions, this revealed significant impacts of GAD on regions implicated in goal‐directed activity (Group‐by‐Task Condition interactions; see below). However, no indications of specific heightened responses to threat were seen (i.e., no regions showing significant Group‐by‐Valence interactions). All other significant results are listed in Table S1.

#### 3.3.1 Group‐by‐Task Condition interactions

**Figure 2:**
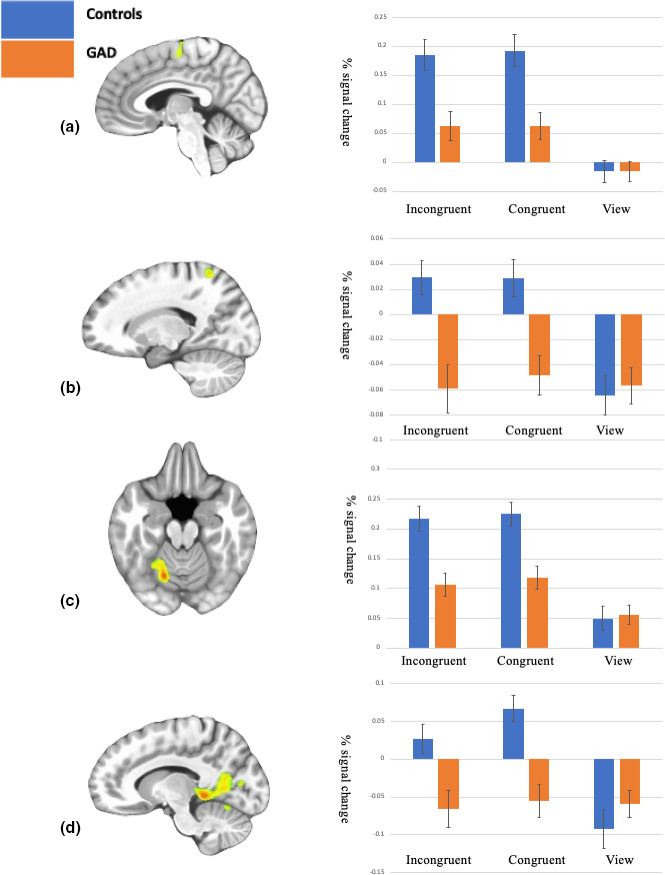
Interactions of Group‐by‐Task Condition. Patients with GAD showed decreased activation to both Congruent and Incongruent trials relative to the View trials compared to the healthy comparison individuals in (a); right dmPFC (*x*, *y*, *z* = 2, =−13, 65 (b) right postcentral gyrus/ precuneus (*x*, *y*, *z* = −49, −4, 23); (c) left culmen (*x*, *y*, *z* = −16, −58, −16); (d) left posterior cingulate gyrus/culmen (*x*, *y*, *z* = −7, 40, −1) (*p* < .001 for each)

The Group‐by‐Task Condition interaction identified regions including bilateral precentral gyrus, dmPFC, precuneus, and posterior cingulate cortex; see Table 2. In all 8 regions identified in the interaction, healthy individuals showed significantly greater activation compared to participants with GAD to both Congruent relative to View (*F* range 11.69–24.97; *p* < .001 for all) and Incongruent relative to View trials (*F* range = 14.66–33.76; *p* < .001 for all). This effect was driven by group differences to the Congruent and Incongruent trials rather than the View trials; Figure 2. Whereas there was no significant group difference to the View trials within any of the 8 regions (*F* range 0.00–2.27; ns), participants with GAD showed significantly less activation compared to the comparison individuals within all 8 regions to both Congruent (*F* range 6.00–18.90; *p* < range .05–.001) and Incongruent trials (except cuneus) (*F* range 7.10–18.46; *p* range .05–.001).

**Table 2:** Significant areas of activation from the 2 (Group: Participants with GAD, participants without GAD) × 3 (Task Condition: Incongruent, Congruent, View) × 3 (Valence: Negative, Neutral, Positive) ANOVA

REGION	BA	Voxels	*X*	*Y*	*Z*	*F*‐value	eta
R Precentral Gyrus	6	52	29	−16	59	16.54	0.263
L Precentral Gyrus	4	36	−19	−22	62	12.24	0.254
L Postcentral gyrus/Precuneus	6	21	−49	−4	23	11.80	0.286
R dmPFC	6	26	2	−13	65	10.30	0.226
L Posterior cingulate gyrus/culmen	30	344	−7	40	−1	15.56	0.228
R Cuneus	18	75	11	−73	20	12.26	0.324
R Precuneus	7	22	17	−52	56	10.50	0.205
L Cerebellum		63	−16	−58	−16	16.50	0.149

Note

coordinates based on the Tournoux & Talairach standard brain template, BA = Brodmann's Area, R = Right, L = Left.

Activations are effects observed in whole‐brain analyses significant at *p* < .001, corrected for multiple comparisons (significant at *p* < .05).

##### Follow‐up analyses

1. ***Excluding participants using medications:*** Given the potential influences of medication use, the group‐based ANOVA above was rerun excluding participants using respectively stimulants, antidepressant, and antipsychotics (See Table 1 ) Regions identified via the Group‐by‐Task Condition were proximal to those seen in the main analysis (Tables S2–S4 **)**.
2. ***Excluding participants with MDD:*** Given the comorbidity of GAD with MDD, the group‐based ANOVA above was rerun excluding participants with MDD (See Table 1). Regions identified via the Group‐by‐Task Condition were proximal to those seen in the main analysis (Table S5). In addition, comparisons between the participants with only GAD and those with GAD & MDD are included in the Supplemental Material.

## 4 DISCUSSION

The goal of this study was to investigate the responsiveness of neural systems engaged in responding to emotional stimuli and top‐down attention during task‐related processing in the presence of emotional distracters in adolescents with GAD. In line with predictions, adolescents with GAD showed reduced activity during task trials, relative to healthy adolescents, within regions implicated in motor (dorsomedial/bilateral precentral gyri and cerebellum) and attentional responding (posterior cingulate cortex, cuneus, and precuneus/superior parietal cortex). However, there were no indications of significantly heightened, or reduced, neural responsiveness to emotional stimuli in the adolescents with GAD.

The previous literature with respect to emotional/threat responsiveness is highly inconsistent with reports of adult patients with GAD showing, relative to comparison healthy individuals, increased (e.g., Buff et al., 2017), comparable (e.g., Whalen et al., 2008) or decreased threat responsiveness (e.g., Blair et al., 2008, 2012). Given our previous results with *adults* with GAD on this task (Blair et al., 2012), we predicted that adolescents with GAD might show evidence of reduced responsiveness to emotional stimuli within the amygdala. However, this prediction was not confirmed. There were no significant group differences in BOLD responses to emotional stimuli in the current study (i.e., no regions showed Group‐by‐Valence interactions). There was, however, a significant Group‐by‐Valence interaction in the RT data; adolescents with GAD showed less slowing of RTs to task trials in the context of positive/negative distracters relative to neutral distracters relative to healthy participants. It is notable that previous work has shown that this slowing is exaggerated in patients with an anxiety disorder consistently associated with heightened threat responsiveness—post‐traumatic stress disorder (Vythilingam et al., 2007). The behavioral result at least is thus consistent with the suggestion that consistent worry in individuals with GAD may interfere with ongoing processing, including emotional processing (e.g., Blair et al., 2008; Ellis & Hudson, 2010).

In line with predictions, a series of regions showed significant Group‐by‐Task Condition interactions. These regions included dorsomedial and lateral frontal cortex, bilateral precentral gyri, cerebellum, posterior cingulate cortex, cuneus and precuneus/superior parietal cortex. These are all regions implicated in response control/motor responding (bilateral precentral gyri and cerebellum; e.g., Scott, 2004) and/or cognitive control/attention (dorsomedial and lateral frontal cortex, posterior cingulate cortex, cuneus and precuneus/superior parietal cortex; see Leech & Sharp, 2014; Remington et al., 2020; Vogt, 2019). As seen in previous literature, the healthy comparison participants in the current study showed greater responses within these regions to task relative to view trials (see Figure 2). In contrast, the adolescents with GAD showed significantly less recruitment of these regions during task trials. Notably, the region of precuneus/superior parietal cortex that showed reduced activity in the adolescents with GAD in the current study was proximal to that showing reduced activity in the earlier work with adults with GAD using this task (Blair et al., 2012).

Moreover, and complimenting these BOLD data, the adolescents with GAD were significantly more inaccurate on this task relative to comparison adolescents. These findings are compatible with previous work reporting reduced executive function performance in patients with GAD (Butters et al., 2011; Hallion et al., 2017; Stefanopoulou et al., 2014; Tempesta et al., 2013). Because of this reduced performance, the claim has been made that consistent worry in individuals with GAD interferes with ongoing processing—not only emotional processing but also executive functioning/task performance (e.g., Blair et al., 2008; Ellis & Hudson, 2010). Of course, this position remains unsatisfactory. Task‐based analyses may be less useful in identifying the pathology associated with worry as opposed to the consequences of this worry.

Two caveats should be considered with respect to the current results. First, some participants with GAD were medicated. However, 3 follow‐up group‐based ANOVAs, excluding participants being medicated with stimulants, antidepressant, or antipsychotics, revealed group differences with respect to regions recruited during task performance that were proximal to the main analysis. As such, we do not believe group differences in medication can account for the current results. Second, a number of the participants with GAD presented with comorbid MDD. This is consistent with previous work where the comorbidity of GAD with MDD is high (Remes et al., 2018; Shen et al., 2018). It is thus possible that the current results might represent the psychopathology of MDD rather than that of GAD. Importantly, however, a follow‐up group‐based ANOVAs, excluding participants with MDD again revealed group differences with respect to regions recruited during task performance that were proximal to the main analysis.

In conclusion, in the current study, participants with GAD showed reduced interference by emotional distracters in their behavioral performance (with respect to RT) and increased errors relative to healthy comparison adolescents. In addition, they showed reduced recruitment of a number of regions implicated in response control/motor responding and/or cognitive control/attention during task trials. Importantly, removal of participants taking stimulants, SSRIs, antipsychotics, or had MDD did not significantly change our findings after follow‐up analyses. We hypothesize that these reflect secondary consequences of the increased worry shown by patients with GAD (c.f., Blair et al., 2008; Ellis & Hudson, 2010). However, it is possible that these impairments reflect risk factors for the emergence of worry.

## CONFLICT OF INTEREST

The authors have no conflicts of interest to disclose.

## AUTHOR CONTRIBUTIONS

KSB and JBL are responsible for the data analysis. They both had full access to all the data in the study and take responsibility for the integrity of the data and the accuracy of the data analysis. JK, KM, and SW contributed to the acquisition and critical revision of the manuscript. JBL, JA, MD, RJR, and KSB contributed to interpretation of the data and critical revision of the manuscript for important intellectual content.

### Peer Review

The peer review history for this article is available at https://publons.com/publon/10.1002/brb3.1994.

## Supporting information

Table S1‐S7

## Data Availability

The data that support the findings of this study are available from the corresponding author upon reasonable request. The data are not publicly available due to IRB restrictions.
