# Supplementary material for: Reduced top‐down attentional control in adolescents with generalized anxiety disorder
Source: Brain Behav. 2020 Dec 25;11(2):e01994. doi: 10.1002/brb3.1994 (PMC7882153; doi:10.1002/brb3.1994)
Supplement: Supplementary file 1 — Table S1‐S7 [file BRB3-11-e01994-s001.docx]

Supplemental Table 1: Significant areas of activation from the 2 (Group: Participants with GAD, participants without GAD) x 3 (Task Condition: Incongruent, Congruent, View) x 3 (Valence: Negative, Neutral, Positive) ANOVA not reported in the main Table 1. Activations are effects observed in whole brain analyses significant at p<0.001 corrected for multiple comparisons (significant at p<0.05).

| REGION | BA | Voxels | X | Y | Z | F-value | eta |
| --- | --- | --- | --- | --- | --- | --- | --- |
| Group |  |  |  |  |  |  |  |
| R Precentral Gyrus | 6 | 27 | 26 | -19 | 59 | 21.14 | .253 |
| L Insula | 13 | 25 | -43 | -19 | -1 | 18.89 | .285 |
|  |  |  |  |  |  |  |  |
| Valence |  |  |  |  |  |  |  |
| RL Culmen/Fusiform Gyrus/ Occipital Gyrus/ Temporal Gyrus | 37 | 2799 | 41 | -43 | 19 | 99.1 | .572 |
| R Parahippocampal Gyrus | 36 | 98 | 29 | -40 | -7 | 22.25 | .278 |
| R Middle Frontal Gyrus | 9 | 90 | 41 | 11 | 29 | 16.55 | .237 |
| L Parahippocampal Gyrus | 36 | 70 | -28 | -40 | -7 | 22.56 | .295 |
| L Middle Frontal Gyrus | 47 | 58 | -31 | 32 | -4 | 22.6 | .218 |
| R Middle Frontal Gyrus | 47 | 43 | 35 | 32 | -4 | 22.6 | .238 |
| R Parahippocampal Gyrus/ Amygdala | 28 | 40 | 20 | -4 | -13 | 21.4 | .282 |
| L Parahippocampal Gyrus/ Amygdala | 28 | 31 | -19 | -4 | -10 | 16.69 | .226 |
| R Middle Frontal Gyrus | 6 | 24 | 23 | 2 | 47 | 11.2 | .167 |
| L Inferior Frontal Gyrus | 46 | 20 | -43 | 26 | 17 | 9.494 | .156 |
|  |  |  |  |  |  |  |  |
| Task Condition |  |  |  |  |  |  |  |
| R Cerebellar Tonsil |  | 19856 | 32 | -43 | -31 | 99.3 | .636 |
| L Anterior Cingulate | 32 | 837 | -4 | 38 | -4 | 27.93 | .307 |
| L Inferior Frontal Gyrus | 46 | 257 | -52 | 26 | 14 | 39.31 | .335 |
| L Middle Temporal Gyrus | 22 | 248 | -64 | -31 | 5 | 18.39 | .233 |
| L Angular Gyrus | 39 | 220 | -49 | -64 | 32 | 23.68 | .263 |
| R Superior Temporal Gyrus | 22 | 219 | 59 | -37 | 8 | 22.13 | .246 |
| R Medial Frontal Gyrus | 6 | 173 | 5 | -25 | 62 | 33.7 | .251 |
| L Parahippocampal Gyrus/ Amygdala | 34? | 170 | -19 | -4 | -16 | 23.65 | .314 |
| R Inferior Frontal Gyrus | 46 | 152 | 53 | 26 | 14 | 22.15 | .257 |
| R Precentral Gyrus | 4 | 129 | 29 | -25 | 50 | 20.33 | .239 |
| L Cingulate Gyrus | 31 | 65 | -10 | -43 | 35 | 25.09 | .209 |
| L Precentral Gyrus | 4/6 | 63 | -43 | -13 | 38 | 30.24 | .250 |
| R Parahippocampal Gyrus/ Amygdala | 28 | 41 | 20 | -4 | -16 | 23.15 | .265 |
| R Middle Temporal Gyrus | 39 | 35 | 53 | -61 | 23 | 11.39 | .171 |
| L Posterior Cingulate | 29 | 31 | -7 | -49 | 11 | 17.87 | .200 |
| R Insula | 13 | 27 | 38 | -16 | 20 | 24.31 | .246 |
| L Superior Frontal Gyrus | 8 | 26 | -19 | 26 | 50 | 11.24 | .152 |
| L Superior Temporal Gyrus | 28 | 20 | -37 | 17 | -31 | 9.599 | .173 |
|  |  |  |  |  |  |  |  |
| Valence-by-Task Condition |  |  |  |  |  |  |  |
| R Middle Temporal Gyrus | 37/19 | 24 | 47 | -64 | 11 | 6.232 | .098 |
|  |  |  |  |  |  |  |  |

Note: coordinates based on the Tournoux & Talairach standard brain template, BA= Brodmann’s Area, R= Right, L= Left

Supplemental Table 2. Significant areas of activation from the 2 (Group: Participants with GAD, participants without GAD) x 3 (Task Condition: Incongruent, Congruent, View) x 3 (Valence: Negative, Neutral, Positive) ANOVA excluding participants with MDD. Activations are effects observed in whole brain analyses significant at p<0.001 (except *p<0.005), corrected for multiple comparisons (significant at p<0.05).

| REGION | BA | Voxels | X | Y | Z | F-value |
| --- | --- | --- | --- | --- | --- | --- |
| R Precentral Gyrus | 6 | 17 | 29 | -16 | 59 | 11.40 |
| L Precentral Gyrus | 4 | 36 | -19 | -19 | 62 | 9.80 |
| R dmPFC* | 6 | 35 | 5 | -7 | 50 | 8.55 |
| L Posterior Cingulate Gyrus | 30 | 22 | -7 | -40 | -1 | 11.11 |
| R Cuneus | 19 | 153 | 16 | -58 | 2 | 10.83 |
| R Precuneus | 7 | 18 | 20 | -49 | 59 | 9.55 |

Note: coordinates based on the Tournoux & Talairach standard brain template, BA= Brodmann’s Area, R= Right, L= Left

Supplemental Table 3. Significant areas of activation from the 2 (Group: Participants with GAD, participants without GAD) x 3 (Task Condition: Incongruent, Congruent, View) x 3 (Valence: Negative, Neutral, Positive) ANOVA excluding participants on stimulants. Activations are effects observed in whole brain analyses significant at p<0.001, corrected for multiple comparisons (significant at p<0.05).

| REGION | BA | Voxels | X | Y | Z | F-value |
| --- | --- | --- | --- | --- | --- | --- |
| R Precentral Gyrus | 6 | 60 | 29 | -16 | 59 | 17.39 |
| L Precentral Gyrus | 4 | 26 | -19 | -22 | 62 | 10.49 |
| R dmPFC | 6 | 35 | 2 | -13 | 65 | 12.62 |
| L Posterior Cingulate Gyrus | 30 | 339 | -7 | 40 | -1 | 16.06 |
| R Cuneus | 18 | 72 | 11 | -73 | 20 | 10.80 |
| R Precuneus | 7 | 10 | 17 | -52 | 56 | 9.364 |
| L. Cerebellum (declive). |  | 72 | -16 | -58 | -16 | 17.17 |

Note: coordinates based on the Tournoux & Talairach standard brain template, BA= Brodmann’s Area, R= Right, L= Left

Supplemental Table 4. Significant areas of activation from the 2 (Group: Participants with GAD, participants without GAD) x 3 (Task Condition: Incongruent, Congruent, View) x 3 (Valence: Negative, Neutral, Positive) ANOVA excluding participants who were on anti-psychotics. Activations are effects observed in whole brain analyses significant at p<0.001, corrected for multiple comparisons (significant at p<0.05).

| REGION | BA | Voxels | X | Y | Z | F-value |  |
| --- | --- | --- | --- | --- | --- | --- | --- |
| R Precentral Gyrus | 6 | 46 | 29 | -19 | 62 | 16.39 |  |
| L Precentral Gyrus | 4 | 28 | -19 | -19 | 65 | 9.793 |  |
| R dmPFC | 6 | 26 | 2 | -13 | 65 | 11.53 |  |
| L Posterior Cingulate Gyrus | 30 | 295 | -10 | -37 | -1 | 14.94 |  |
| R Cuneus | 18 | 54 | 11 | -73 | 20 | 11.30 |  |
| R Precuneus | 7 | 12 | 17 | -52 | 56 | 8.165 |  |
| L. Cerebellum (declive) |  | 91 | -16 | -58 | -16 | 18.68 |  |

Note: coordinates based on the Tournoux & Talairach standard brain template, BA= Brodmann’s Area, R= Right, L= Left

Supplemental Table 5. Significant areas of activation from the 2 (Group: Participants with GAD, participants without GAD) x 3 (Task Condition: Incongruent, Congruent, View) x 3 (Valence: Negative, Neutral, Positive) ANOVA excluding participants taking SSRIs. Activations are effects observed in whole brain analyses significant at p<0.001 (except *p<0.005), corrected for multiple comparisons (significant at p<0.05).

| REGION | BA | Voxels | X | Y | Z | F-value |  |
| --- | --- | --- | --- | --- | --- | --- | --- |
| R Precentral Gyrus | 6 | 39 | 29 | -16 | 59 | 11.26 |  |
| L Precentral Gyrus | 4 | 15 | -19 | -25 | 59 | 9.53 |  |
| R dmPFC* | 6 | 35 | 2 | -13 | 65 | 6.39 |  |
| L Posterior Cingulate Gyrus | 30 | 21 | -7 | -40 | -4 | 10.03 |  |
| R Cuneus | 18 | 42 | 2 | -70 | 29 | 9.76 |  |
| R Precuneus | 7 | 43 | 20 | -46 | 56 | 14.29 |  |

Note: coordinates based on the Tournoux & Talairach standard brain template, BA= Brodmann’s Area, R= Right, L= Left

Supplemental Table 6: Subject characteristics.

|  | GAD (N=20) | | GAD & MDD (N=15) | |  | |  | |  |
| --- | --- | --- | --- | --- | --- | --- | --- | --- | --- |
|  | Mean | sd | Mean | sd | | F(1,33) | | p | |
| Age | 16.25 | 1.48 | 15.60 | 1.40 | | 1.72 | | 0.20 | |
| Sex | 10 M/ 10 F |  | 5 F/ 10 M |  | | 0.94 | | 0.34 | |
| IQ | 104.65 | 10.32 | 101.80 | 16.67 | | 0.39 | | 0.54 | |
| SCARED Total | 34.40 | 14.27 | 43.73 | 14.22 | | 3.68 | | 0.06 | |
| SCARED_PN | 8.55 | 6.58 | 12.13 | 4.50 | | 3.28 | | 0.08 | |
| SCARED_GD | 11.35 | 3.45 | 12.87 | 4.64 | | 1.23 | | 0.28 | |
| SCARED_SP | 5.40 | 3.33 | 6.47 | 4.47 | | 0.66 | | 0.42 | |
| SCARED_SC | 6.95 | 2.89 | 8.73 | 3.17 | | 3.00 | | 0.09 | |
| SCARED_SH | 2.15 | 2.03 | 3.53 | 2.20 | | 3.70 | | 0.06 | |
| MFQ | 16.71 | 13.99 | 31.33 | 16.42 | | **7.40** | | 0.01 | |
| CDI | 12.50 | 5.24 | 21.79 | 6.87 | | **17.56** | | 0.00 | |
| Antipsychotic meds | 2 |  | 2 |  | | 0.09 | | 0.77 | |
| Stimulants | 4 |  | 1 |  | | 1.22 | | 0.28 | |
| SSRIs | 6 |  | 6 |  | | 0.36 | | 0.55 | |

Key to table: SCARED: Screen for Child Anxiety Related Disorders; MFQ: Mood and Feelings Questionnaire; CDI: Child Depression Inventory. PN=Panic Disorder or Significant Somatic Symptoms; GD=GAD; SP=Separation Anxiety Disorder; SC=Social Anxiety Disorder; SH=Significant School Avoidance

**Contrasting the participants with only GAD and those with GAD & MDD**

Participant characteristics: Fifteen of the 35 participants with GAD were also comorbid for MDD. These groups did not differ significantly in age, sex or IQ (see Supplemental Table 6) or an anxiety scales from the SCARED. However, the participants with GAD & MDD showed significantly greater depression symptomatology as indexed by the Child Depression Inventory and the Mood and Feelings Questionnaire (see Supplemental Table 6).

Behavioral data: Two 2 (Group: GAD, GAD/MDD) by 3 (Emotion: Negative, Positive, Neutral) by 2 (Condition: Congruent, Incongruent) ANOVAs were conducted on the accuracy and RT data. Both revealed task effects (F(1,33)=25.14 & 55.98 for accuracy and RT respectively). However, there were no significant main effects of Group or interactions with Group (in all cases F<1.5, p=0.24 or greater).

BOLD response data: A 2 (Group: GAD, GAD/MDD) by 3 (Emotion: Negative, Positive, Neutral) by 3 (Condition: Congruent, Incongruent, View) ANOVA was conducted on the BOLD response data. There were regions showing comparable main effects of Emotion and Condition as those described in Supplemental Table 1. However, no regions showed main effects of Group or Emotion/Condition interactions with Group.

Supplemental Table 7: Behavioral Data of Reaction Times and Accuracy by Task Condition

|  | **Reaction Times (in ms)** | |
| --- | --- | --- |
| **Condition** | **GAD** | **Without GAD** |
| Congruent Negative | 784.95 | 748.24 |
| Incongruent Negative | 838.49 | 823.28 |
| Congruent Neutral | 792.83 | 746.36 |
| Incongruent Neutral | 834.22 | 800.31 |
| Congruent Positive | 767.59 | 762.99 |
| Incongruent Positive | 840.73 | 827.88 |
|  | **Accuracy (%)** | |
| **Condition** | **GAD** | **Without GAD** |
| Congruent Negative | 82.41 | 89.88 |
| Incongruent Negative | 74.19 | 85.84 |
| Congruent Neutral | 81.16 | 90.72 |
| Incongruent Neutral | 75.09 | 85.03 |
| Congruent Positive | 82.16 | 91.53 |
| Incongruent Positive | 76.25 | 87.50 |
